# Antiproliferative and apoptotic effects of β-elemene on human hepatoma HepG2 cells

**DOI:** 10.1186/1475-2867-13-27

**Published:** 2013-03-14

**Authors:** Zhi-Jun Dai, Wei Tang, Wang-Feng Lu, Jie Gao, Hua-Feng Kang, Xiao-Bin Ma, Wei-Li Min, Xi-Jing Wang, Wen-Ying Wu

**Affiliations:** 1 Department of Oncology, the Second Affiliated Hospital of Xi’an Jiaotong University, Xi’an 710004, China; 2 Department of life science, Shaanxi Normal University, Xi’an, 710061, China; 3 Department of Nephrology, the Second Affiliated Hospital of Xi’an Jiaotong University, Xi’an 710004, China; 4 Department of Pharmacology, the Second Affiliated Hospital of Xi’an Jiaotong University, Xi’an 710004, China

**Keywords:** Hepatoma, β-elemene, Apoptosis, Fas, FasL

## Abstract

**Background:**

β-elemene, a natural sesquiterpene extracted from the essential oils of *Curcuma aromatica Salisb*, has been shown to be effective against a wide range of tumors. In this study, the antitumor effect of β-elemene on a human hepatoma cell line, HepG2, and the mechanism involved have been investigated.

**Methods:**

MTT assay was used to determine the growth inhibition of hepatoma HepG2 cells *in vitro*. Apoptosis of HepG2 cells were demonstrated by fluorescence microscope with Hoechst 33258 staining and flow cytometry with Annexin V-FITC/PI double staining. Flow cytometry was performed to analyze the cell cycle distribution of HepG2 cells. The mRNA and protein expression of Fas and FasL were measured by RT-PCR and Western blot analysis.

**Results:**

MTT results showed that β-elemene could inhibit the proliferation of HepG2 cells in a time- and dose- dependent manner. Our results showed β-elemene had positive effect on apoptosis through fluorescence microscope and flow cytometry assay. Furthermore, β-elemene could induce the cell cycle arrest of the HepG2 cells in the G_2_/M phase. Fas and FasL expression were obviously increased after β-elemene treatment in both mRNA and protein level.

**Conclusion:**

The present study indicates that β-elemene can effectively inhibit proliferation and induce apoptosis in hepatoma HepG2 cells, and the apoptosis induction is related with up-regulating of Fas/FasL expression.

## Background

Hepatocellular carcinoma (HCC) is the fifth most common cancer and the second leading cause of cancer-related deaths worldwide [1]. The causes of HCC includes HBV or HCV infection, alcohol intake, smoking and aflatoxin. In China, HBV is the most important risk factor for the development of HCC [2].

The treatment of patients with HCC is particularly challenging because of the array of patient-specific (medical comorbidities), tumor-specific (size, number, location, and vascular involvement), and liver-specific (parenchymal reserve) variables that impact our ability to treat patients safely and effectively [3]. Liver resection remains the gold standard for patients with resectable HCC that develops in the setting of normal liver substance. However, most patients with HCC have diseased liver parenchyma and resection in this population is more fraught, with the potential for complications.

Eventually, most patients will receive some forms of chemotherapy in hope of prolonging life. However, the effect of chemotherapy is not satisfied, and the side effects are often difficult to tolerate. Sorafenib is the first molecular inhibitor to be approved by the FDA for the treatment of advanced HCC. While being considered an advance over conventional chemotherapy, sorafenib only shows approximate 3 months survival advantage over the nontreated group [4,5]. Prior to the arrival of sorafenib, doxorubicin was routinely used as a single drug for advanced HCC, but has shown inefficacy, with a response rate of about 15-20%. Other chemotherapy agents, such as epirubicin, cisplatin, 5-fluorouracil and their combinations, demonstrate even lower efficacy [6]. With that in mind, people have being paying more attention to searching for new antitumor agents from natural products [7]. Many Chinese herbs have been discovered to be potential sources of antitumor drugs [8,9].

It was reported that many Chinese herbs had anticancer properties and induce apoptosis [10]. Three apoptotic pathways have been addressed, including the mitochondrial pathway, death receptor pathway, and endoplasmic reticulum stress-mediated apoptosis pathway [11]. Fas is a member of the death receptor family. Stimulation of Fas leads to induction of apoptotic signals, such as caspase 8 activation, as well as “non-apoptotic” cellular responses, notably NF-κB activation [12]. Fas ligand (FasL) is a type II transmembrane protein and signals through trimerization of FasR, which spans the membrane of the “target” cell. This trimerization usually leads to apoptosis, or cell death [13].

**Figure 1:**
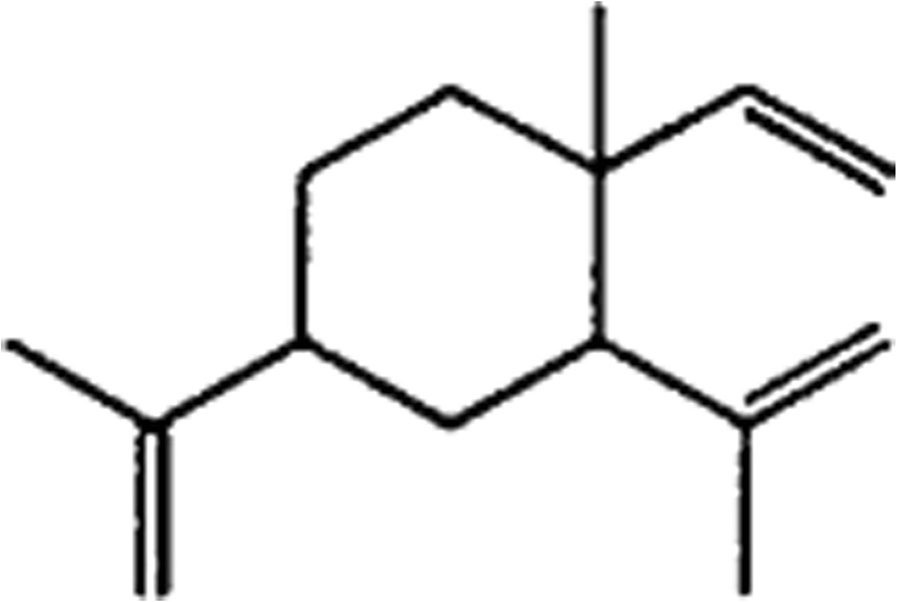
**Chemical structure of β-elemene. **The molecular formula of β-elemene is C15H24 and its molecular weight 204.34.

*Curcuma aromatica Salisb.* is a popular type of traditional Chinese medicine plant whose essential oils are widely used in cancer treatment in China [14]. β-elemene (1-methyl-1-vinyl-2,4-diisopropenyl-cyclohexane), a sesquiterpene extracted from the essential oils of *Curcuma aromatica Salis.*, accounts for 60-72% of elemene in *Curcuma aromatica Salisb*[15]*.* Among the active ingredients, β-elemene is the most widely studied, whereas δ-elemene, furanodiene, furanodienone, curcumol, and germacrone have just recently attracted the attention of researchers [14]. The molecular formula of β-elemene is C15H24 and its molecular weight 204.34. The chemical structure of β-elemene was showed in Figure 1. Meta-analysis results suggest that β-elemene can improve the effect of chemotherapy in lung cancer as an adjunctive treatment [16]. The combined treatment can improve quality of life and prolong survival. In vitro, β-elemene has shown promising anti-cancer effects against a broad spectrum of tumors, such as lung, breast, prostate, cervical, gastric, ovarian cancer and osteosarcoma [17-22]. However, it was few reported in hepatoma cells. The aim of the present study is to investigate the antitumor effect of β-elemene on human hepatoma HepG2 cells and the molecular mechanism involved.

## Methods

### Reagents

Fetal bovine serum (Gibco, USA); RPMI1640 medium (Gibco, USA); 3-(4,5)-dimethylthiahiazo (-z-y1)-3,5-diphenyte-trazoliumromide (MTT) and propidium iodide (PI) were purchased from Sigma Chemical (St. Louis, MO). Annexin V-FITC/PI apoptosis detection kit (Becon Dickinson Facsalibur, USA); RT-PCR kit (ampliqon, Denmark); Trizol (Invitrogen, USA); β-elemene was obtained from Dalian Holley Kingkong Pharmaceutical Co., Ltd (Dalian, China). Anti-Fas and Anti-FasL were purchased from Santa Cruz Biotechnology.

### Cell line and cell culture

The protocol in this study was approved by the Committee on the Ethics of Animal Experiments of Medicine College, Xi’an Jiaotong University (Certificate No. 22-9601018).

Human hepatoma HepG2 cell line was obtained from Cancer Institute of the Fourth Military Medical University (Xi’an, China). Cells were cultured in RPMI 1640 medium containing 10% inactived fetal bovine serum in a humidified atmosphere with 5% CO_2_ incubator at 37°C.

### MTT assay for the proliferation of HepG2 cells

Viability of HepG2 cells was assessed using MTT dye reduction assay (Sigma, USA), which was conducted as described previously [23]. Cells were seeded in a 96-well plate at a density of 1 ×10^4^ cells/well, cultured for 12 h, and then treated with different concentration (0, 40, 80 and 120 μg/mL) β-elemene for 0-96 h. At the end of the treatment, MTT, 50 μg/10 μL, was added and the cells were incubated for another 4 hours. Dimethylsufloxide (DMSO; 200 μL) was added to each well after removal of the supernatant. After shaking the plate for 10 min, cell viability was assessed by measuring the absorbance at 490 nm using an Enzyme-labeling instrument (EX-800 type); all measurements were performed three times. The results represented as the average of three independent experiments done over multiple days.

The growth inhibition was calculated according to the following formula: The Growth Inhibition Ratio (IR*%*) = [(the absorbance of blank control group - the absorbance of experimental group)/the absorbance of blank control group] × 100*%*.

### Detection of morphological apoptosis with Hoechst 33258 staining

HepG2 cells were harvested by centrifugation, washed with PBS and fixed with 1% glutaraldehyde for 1 h at room temperature. Fixed cells were washed with PBS, stained with 200 μM Hoechst 33258 for 10 min, and the changes in the nuclei of cells after stained with Hoechst 33258 were observed using a fluorescence microscope (Olympus, BX-60, Japan).

### Apoptosis detection by flow cytometry (FCM)

Apoptotic cells were differentiated from viable or necrotic ones by combined application of annexin V-FITC and propidium iodide (PI) (BD Biosciences Clontech, USA) [13]. The samples were washed twice and adjusted to a concentration of 1×10^6^ cells/mL with 4°C PBS. The Falcon tubes (12 mm×75 mm, polystyrene round-bottom) were used in this experiment, 100 μL of suspensions was added to each labeled tube, 10 μL of annexin V-FITC and 10 μL PI (20 μg/mL) were added into the labeled tube, incubated for at least 20 min at room temperature in the dark, then 400 μL of PBS binding buffer was added to each tube without washing and analyzed using FCM analysis (BD Biosciences Clontech, USA) as soon as possible (within 30 min). This assay was done triplicate.

### Cell cycle analysis

HepG2 cells were incubated at 5 × 10^5^ cells/well in 6-well plates with serum-free RPMI1640 medium for 12 h, then treated with a homologous drug for 48 h. The detached and attached cells were harvested and fixed in 70% ice-cold ethanol at -20°C overnight. After fixation, cells were washed with PBS, resuspended in 1 mL PBS containing 1 mg/mL RNase (Sigma) and 50 μg/mL PI (Sigma), and incubated at 37°C for 30 min in the dark. Samples were then analyzed for DNA content by flow cytometry (Beckman, USA), and cell cycle phase distributions were analyzed with the Cell Quest acquisition software (BD Biosciences).

### Reverse transcription-polymerase chain reaction (RT-PCR)

HepG2 cells were seeded in 6 cm culture capsules and treated with various concentration of β-elemene (10, 20 and 40 μg/mL) separately for 48 h. Each group contained 3 culture capsules. As previously described [24], cells collected at specified time to extract total RNA using the Trizol reagent following the manufacturer’s instructions. 1 μg RNA synthetized cDNA through reverse transcriptase undergo listed below condition: 70°C 5 min, 42°C extended for 60 min, 95°C enzyme inactivated for 3 min and 4°C terminated reaction. Synthetical cDNA as template to carry out polymerase chain reaction. Fas primer sequence: 5^′^ TTCTGCCATAAGCCCTGTC -3^′^ (forward) and 5^′^ TTGGTGTTGCTGGTGAGT -3^′^ (reverse), amplification fragment was 320 bp, renaturation temperature was 55°C (cycling 25 times). FasL primer sequence: 5^′^ TTCAGCTCTTCCACCTACAG -3^′^ (forward) and 5^′^ ACATTCTCGGTGCCT GTAAC-3^′^ (reverse), amplification fragment was 599 bp. β-actin primer sequence was 5^′^ GGTGCTGAGTATGTCGTGGAG -3^′^ (forward), 5^′^ ATGCA GGGATGATGTTCTAGG -3^′^ (reverse), amplification fragment was 390 bp. Renaturation temperature was 55°C (cycling 20–25 times). Amplification condition was below: pre-denaturized for 3 min at 95°C, denaturized for 30 s at 95°C, renaturated for 30 s at 55°C and extended for 30 s at 72°C. PCR product was detected on agarose gel electrophoresis and ethidium bromide imaging system was used to make density index analysis. The expression intensity of destination gene mRNA was denoted with the ratio of the photodensity of the RT-PCR products of destination gene and β-actin.

### Western blot analysis

HepG2 cells were treated with various concentration of β-elemene (10, 20 and 40 μg/mL) for 48 h. As previously described [23], cells were lysed in a sample buffer followed by denaturation. Protein concentrations were determined using the PIERCE BCA protein assay kit and equal amounts of protein (50 μg) were subjected to SDS-PAGE on 10% gel. The proteins were then electrophoretically transferred to nitrocellulose membranes. The membranes were blocked with 5% skimmed milk, respectively incubated with anti-Fas, anti-FasL (1:500; Santa Cruz Biotechnology) at 4°C overnight. And then followed by incubation in goat antimouse secondary antibody conjugated with horseradish peroxidase (1:1000; Santa Cruz Biotechnology). Equal loading of each lane was evaluated by immunoblotting the same membranes with β-actin antibodies after detachment of previous primary antibodies. Photographs were taken and optical densities of the bands were scanned and quantified with the Gel Doc 2000 (Bio-Rad).

### Statistical analysis

Data were expressed as Mean ± SEM. Statistical analysis was performed with one-way analysis of variance (ANOVA) test using the statistical software SPSS 13.0. *P* values less than 0.05 were considered statistically significant.

## Results and discussion

### β-elemene inhibits proliferation in hepatoma HepG2 cells

β-elemene has antiproliferative effect on several cancer cell lines. It could inhibit the growth of laryngeal cancer HEp-2 cells in vitro in a dose- and time-dependent manner. *In vivo*, the growth of HEp-2 cell-transplanted tumors in nude mice was inhibited by intraperitoneal injection of β-elemene [25]. Bao et al. also reported that β-elemene could inhibit the growth of mouse hepatoma H22 tumor cells in a time- and dose-dependent manner [26]. Furthermore, β-elemene can downregulate the levels of plasma endotoxins, serum TNF-α, and CD14 expression in hepatic fibrosis rats. β-elemene may play an important role in liver tumorigenesis [27].

**Figure 2:**
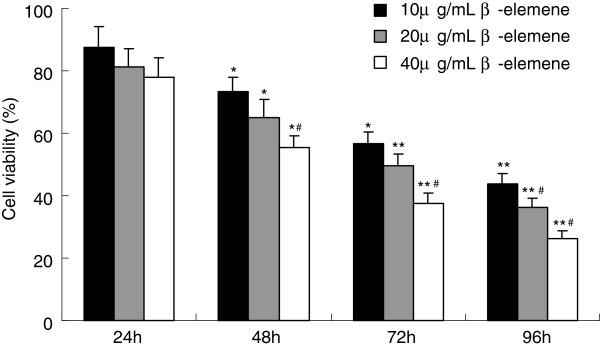
**Growth inhibiting effects of β-elemene on HepG2 cells. **HepG2 cells were treated with different concentrations drug for 0-96 h. Cell viability was determined by MTT method. This assay was performed in triplicate. Dose- and time-dependent inhibition of cell growth could be observed after 96 h (*P*<0.05, ANOVA analysis). ^*^P<0.05, ^**^P<0.01 versus 24 h; ^#^*P*<0.05 versus 10 μg/mL β-elemene group.

In this study, HepG2 cells were treated with different doses of β-elemene. MTT assay was used to examine the anti-proliferative effect of β-elemene on HepG2 cells. The effects of β-elemene (10, 20 and 40 μg/mL) on cell growth after 96 h are shown in Figure 2. The inhibitory rate of β-elemene on cell growth was 73.7 ± 4.4% when treated with high concentrations of β-elemene (40 μg/mL) for 96 hours. MTT assay showed that β-elemene significantly suppressed the proliferation of HepG2 cells, in a dose- and time-dependent manner with cell numbers markedly reduced compared to the control group.

### Measurement of apoptosis of HepG2 cells by Hoechst 33258 staining

The anti-cancer effects of β-elemene are related to the retardation of cell cycle arrest, the induction of apoptosis, and the inhibition of metastasis or tissue invasion [14]. Liu et al. also reported that β-elemene induced protective autophagy and prevented human gastric cancer cells from undergoing apoptosis [20]. β-elemene could induce apoptosis by inhibiting the Hsp90/Raf-1 complex on glioblastoma cells [28].

**Figure 3:**
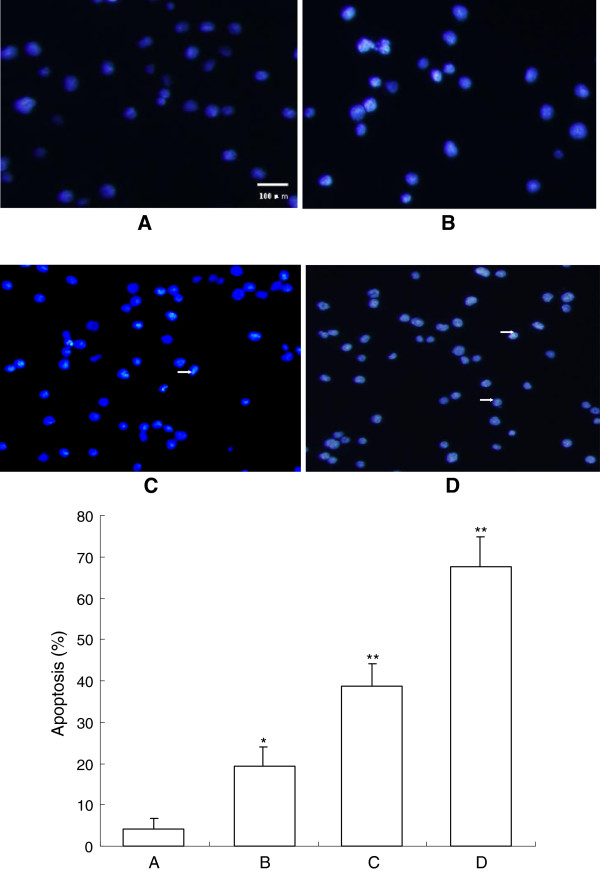
**Cell apoptosis observed by Hoechst 33258 staining using a fluorescence microscope (200×). **After cells were treated with different dose of β-elemene for 72 h, Hoechst 33258 staining was used to observe the apoptotic cells under a BX-60 fluorescence microscope. Values represent mean ± SEM from three independent experiments. **A**: blank control group; **B**: 10 μg/mL β-elemene group; **C**: 20 μg/mL β-elemene group; **D**: 40 μg/mL β-elemene group. ^*^P<0.05, ^**^P<0.01 versus blank control group. The arrow shows apoptotic cells.

After treatment with different doses of β-elemene for 48 h, HepG2 cells were stained with Hoechst 33258 and observed under a fluorescence microscope (Olympus, BX-60, Japan). The dye stains condense chromatin of apoptotic cells more brightly than chromatin of normal cells. As shown in Figure 3, the morphological changes of cell apoptosis including condensation of chromatin and nuclear fragmentation were observed in the β-elemene treated groups (Figure 3B-D), while few apoptotic cells in the control group (Figure 3A). The percentage of apoptotic cells in the control group and (10, 20 and 40 μg/mL) β-elemene treated groups were 4.25 ± 2.56%, 19.28 ± 4.72%, 38.65 ± 5.52%, 67.54 ± 7.35% respectively. Furthermore, apoptotic cells gradually increased in a dose-dependent manner.

### FCM analysis of cell apoptosis induced by β-elemene

To demonstrate the apoptosis inducing effect of β-elemene, we also used FCM analysis with annexin V-FITC and PI double staining. After treatment with different doses of β-elemene for 72 h, apoptosis induction was observed. Apoptotic cells were differentiated from viable or necrotic ones by combined application of annexin V-FITC and PI. Apoptotic and necrotic cells were distinguished according to annexin V-FITC reactivity and PI exclusion.

**Figure 4:**
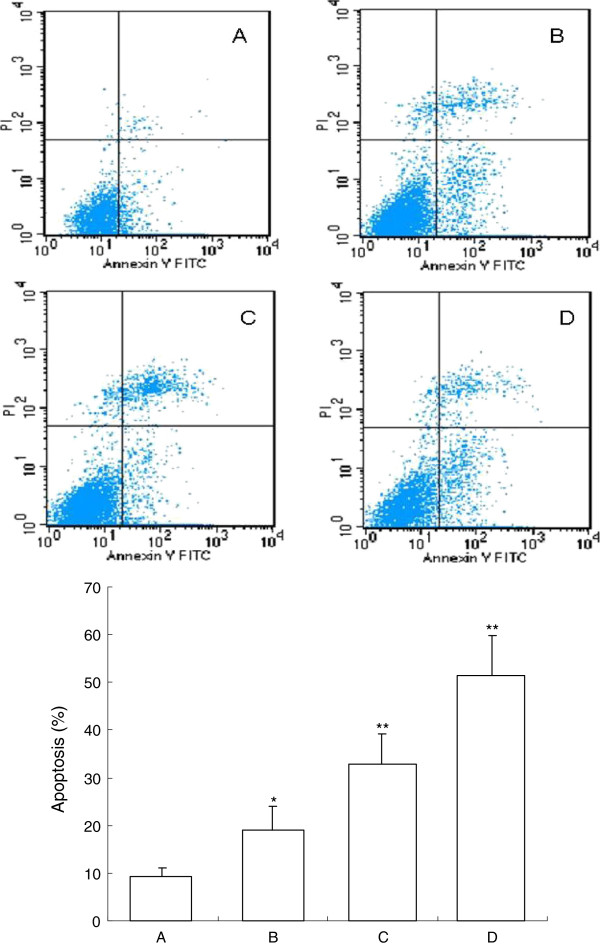
**FCM analysis for apoptosis after treatment by Annexin V-FITC and PI staining on HepG2 cells. **After treatment with different doses of β-elemene for 48 h, apoptosis induction was observed. Apoptotic cells were differentiated from viable or necrotic ones by combined application of annexin V-FITC and PI. This assay was performed in triplicate. **A**: blank control group; **B**: 10 μg/mL β-elemene group; **C**: 20 μg/mL β-elemene group; **D**: 40 μg/mL β-elemene group. ^*^P<0.05, ^**^P<0.01 versus control group.

As shown in Figure 4, in normal control group, there were almost normal cells, rarely viable apoptotic cells; while in β-elemene groups, the rate of apoptotic cells was gradually increased along with increasing concentrations of β-elemene. The rate of apoptosis in normal control and (10, 20 and 40 μg/mL) β-elemene groups were (9.24 ± 1.78)%, (18.92 ± 5.16)%, (32.83±6.25)% and (51.33 ± 8.46)%, respectively. Furthermore, apoptotic cells gradually increased in a dose-dependent manner.

### Effects of β-elemene on the cell cycle distribution by flow cytometry

It was reported that β-elemene induced a persistent block of cell cycle progression at the G_2_/M phase in ovarian cancer A2780 and A2780/CP cells [29]. In glioblastoma cell lines, β-elemene could inhibit the proliferation and arrest cell-cycle in G_0_/G_1_ phase [30]. β-elemene had inhibitory effects on cell growth and blocked NSCLC cells at G_2_/M phase, the arrest being accompanied by decreases in the levels of cyclin B1 and increases in the levels of p27(kip1) [17,21]. Li et al. also reported β-elemene induced cell cycle arrest and apoptotic cell death in human NSCLC H460 cells [19]. However, in another NSCLC cell line, A549 cells, β-elemene inhibited the viability of A549 cells by inducing apoptosis without altering the cell cycle distribution [31].

**Figure 5:**
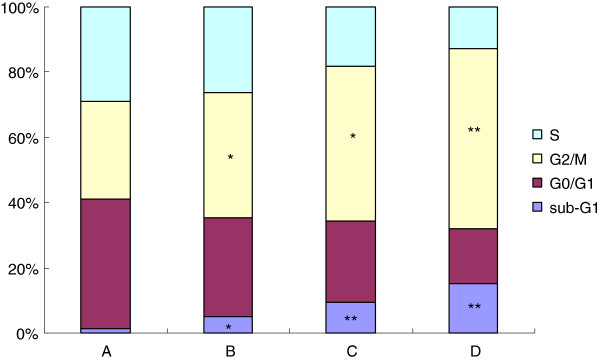
**Effects of β-elemene on the cell cycle of HepG2 cells by FCM. **The cell cycle distributions in HepG2 cells were determined by PI staining and FCM analysis after treated with 0-40 μmol/L β-elemene for 48 h. **A**: blank control group; **B**: 10 μg/mL β-elemene group; **C**: 20 μg/mL β-elemene group; **D**: 40 μg/mL β-elemene group. Results presented were representative of three independent experiments. ^*^P<0.05, ^**^P<0.01 versus control group.

In the present study, the effects of β-elemene on cell cycles were analyzed using FCM. The sub-G_1_ population indicated apoptotic-associated chromatin degradation. The ratios of cell apoptosis in control group and (10, 20 and 40 μg/mL) β-elemene treated groups were 1.47%, 5.21%, 9.37%, 15.25%, respectively. There were significantly difference between β-elemene treated groups and the control group (*P* < 0.05). The percentage of cells in the β-elemene treated groups significantly decreased at the S phase and G_0_/G_1_ phase, simultaneous increased at the G_2_/M phase. These results suggest that β-elemene can induce cell cycle arrest at the G_2_/M phase in HepG2 cells (Figure 5).

### β-elemene promotes Fas and FasL mRNA expression in HepG2 cells with RT-PCR assay

Fas receptor, an important cell surface receptor protein of the TNF receptor family known also as CD95, which induces apoptosis on binding FasL. Fas (CD95/APO-1)/ FasL system is one of the major apoptotic pathways and plays an important role in maintenance of cell colony, elimination of malignant transformation cells and regulation of cell apoptosis [32,33]. Fas and FasL are mainly expressed in cell membrane and the combination of Fas and FasL leads to cell apoptosis [33]. Both Fas and FasL are expressed in most cancer cells. When external FasL combine with Fas expressed on the surface of tumor cells, a complex will form to start the signal transduction of cell apoptosis, and apoptotic signal can be transferred to caspases which are the executors of cell apoptosis by cytoplasmic signal proteins [33]. Then the activated caspases degrade the specific substrates, and finally the activated capases-3 causes DNA breakage leading to apoptosis [13]. It was reported that apoptosis induced by β-elemene seems to be initiated through a p53- and Fas-independent pathway via mitochondria in lung cancer cells [34].

**Figure 6:**
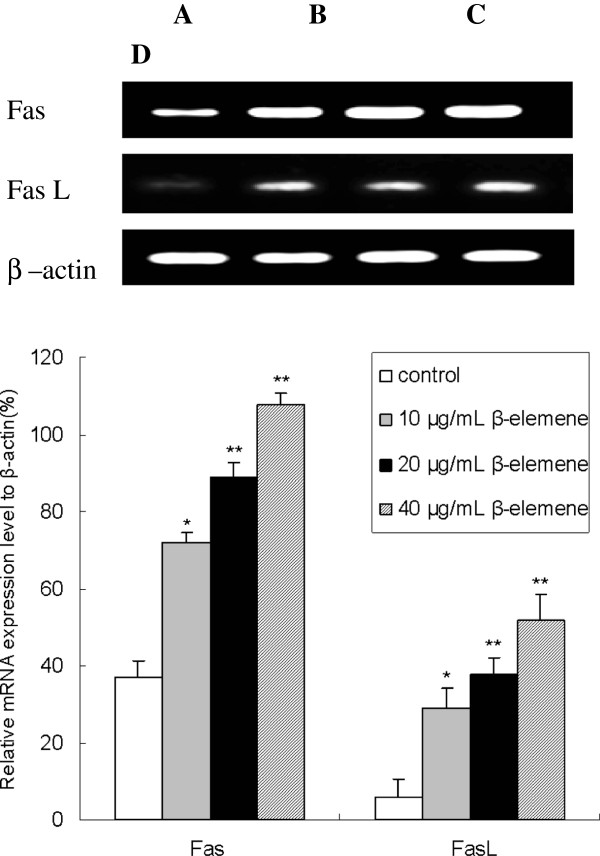
**Effect of β-elemene on Fas and FasL mRNA expression in HepG2 cells. **HepG2 cells were treated with various concentrations of β-elemene (10, 20 and 40 μg/mL) for 48 h, and then the mRNA expression of Fas and FasL were measured by RT-PCR assay. Values represent means ± SEM. **A**: blank control group; **B**: 10 μg/mL β-elemene group; **C**: 20 μg/mL β-elemene group; **D**: 40 μg/mL β-elemene group. This assay was done triplicate (*P<0.05, **P<0.01 versus the control group).

To confirm whether β-elemene induces apoptosis via increasing Fas/FasL expression, expression of Fas and FasL in HepG2 cells were identified by RT-PCR assay. As shown in Figure 6, there were significant increase in both Fas and FasL mRNA expression when treated with 10, 20 and 40 μg/mL β-elemene in comparison with the control group (*P*<0.05). Furthermore, the mRNA expression of Fas and FasL in 40 μg/mL β-elemene group were enhanced up to 192% and 550% respectively, this enhancement effect was in a dose-dependent manner (*P*<0.05).

### β-elemene increases Fas and FasL protein expression with Western blot analysis

**Figure 7:**
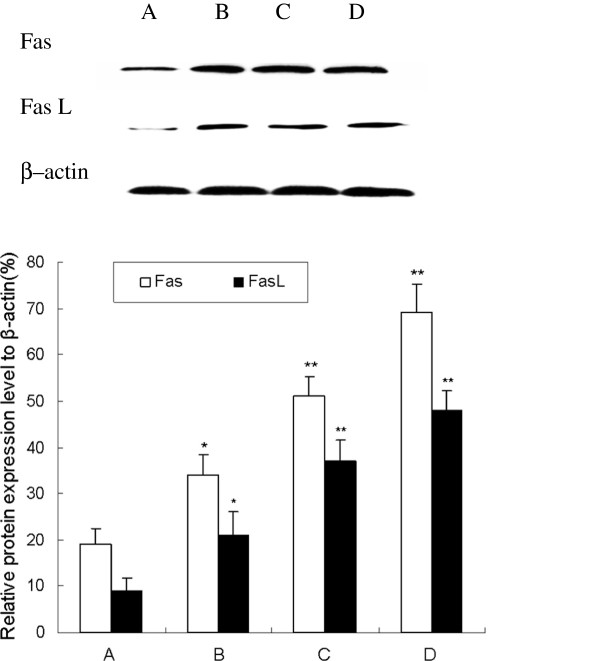
**Effect of β-elemene on Fas and FasL protein expression in HepG2 cells. **HepG2 cells were treated with various concentrations of β-elemene (10, 20 and 40 μg/mL) for 48 h. The protein expression of Fas and FasL were determined by Western blot analysis. **A**: blank control group; **B**: 10 μg/mL β-elemene group; **C**: 20 μg/mL β-elemene group; **D**: 40 μg/mL β-elemene group. Values represent means ± SEM. This assay was done triplicate (*P<0.05, **P<0.01 versus blank control group).

As mentioned above, the Fas and FasL mRNA expression were increased after β-elemene treatment. To examine whether β-elemene increased the expression of Fas and FasL in protein level, we tested the expression with Western blot analysis. As shown in Figure 7, Western blot results showed that both Fas and FasL protein expression significantly increased in β-elemene treated group comparing with the control group (*P*<0.05). Furthermore, the protein expression of Fas and FasL in 40 μg/mL β-elemene group were enhanced 263% and 433%, this enhancement effect was in a dose-dependent manner (*P*<0.05).

## Conclusion

In conclusion, MTT assay showed that β-elemene could inhibit the proliferation of HepG2 cells *in vitro* in a time- and dose- dependent manner. Furthermore, β-elemene could induce apoptosis and cell cycle arrest at the G_2_/M phase in HepG2 cells. The present study suggests that β-elemene can effectively inhibit proliferation and induce apoptosis in hepatoma cells, and the apoptosis induction is related with up-regulating of Fas/FasL expression. However, further studies are necessary to clarify the detailed mechanism involved in the antitumor effects of β-elemene.

## Competing interests

The authors declare that they have no competing interests.

## Authors’ contributions

DZJ, WXJ and WWY designed the research. DZJ, TW, LWF, GJ, and MXB performed the experiments throughout this research. MWL, KHF and WXJ contributed to the reagents, and participated in its design and coordination. TW and LWF analyzed the data; DZJ and GJ contributed to the writing of the manuscript. Co-first authors: DZJ, TW, LWF and GJ. All authors have read and approved the final manuscript.
